# Sumoylation of the DNA polymerase ε by the Smc5/6 complex contributes to DNA replication

**DOI:** 10.1371/journal.pgen.1008426

**Published:** 2019-11-25

**Authors:** Xiangzhou Meng, Lei Wei, Xiao P. Peng, Xiaolan Zhao

**Affiliations:** 1 Molecular Biology Department, Memorial Sloan Kettering Cancer Center, New York, New York, United States of America; 2 Tri-Institutional MD-PhD Program of Weill Cornell Medical School, Rockefeller University, and Sloan-Kettering Cancer Center, New York, New York, United States of America; University of California San Francisco, UNITED STATES

## Abstract

DNA polymerase epsilon (Pol ε) is critical for genome duplication, but little is known about how post-translational modification regulates its function. Here we report that the Pol ε catalytic subunit Pol2 in yeast is sumoylated at a single lysine within a catalytic domain insertion uniquely possessed by Pol2 family members. We found that Pol2 sumoylation occurs specifically in S phase and is increased under conditions of replication fork blockade. Analyses of the genetic requirements of this modification indicate that Pol2 sumoylation is associated with replication fork progression and dependent on the Smc5/6 SUMO ligase known to promote DNA synthesis. Consistently, the *pol2* sumoylation mutant phenotype suggests impaired replication progression and increased levels of gross chromosomal rearrangements. Our findings thus indicate a direct role for SUMO in Pol2-mediated DNA synthesis and a molecular basis for Smc5/6-mediated regulation of genome stability.

## Author summary

DNA replication factors are tightly regulated to ensure genome duplication accuracy and efficiency. Among these factors, the Pol ε replicative polymerase plays a vital role by copying half of the genome every cell cycle. However, little is known about how this critical enzyme is regulated. Here we describe SUMO-based regulation of the catalytic subunit of Pol ε, Pol2. Our data suggest that Pol2 sumoylation occurs during replication elongation, particularly when replication forks encounter template obstacles. This modification is mediated by the conserved Smc5/6 SUMO ligase complex and occurs at a single site within the Pol2 catalytic domain. Several observations suggest that Pol2 sumoylation makes positive contributions to the synthesis of DNA regions enriched with template barriers and helps to prevent large-scale genomic alterations. Our work thus provides new insights into DNA polymerase regulation, specifically the role played by contributions from SUMO and the Smc5/6 complex.

## Introduction

Faithful duplication of the genome is essential for organismal growth and for the prevention of diseases caused by genome instability. The highly conserved four-subunit DNA polymerase epsilon (Pol ε) is a critical enzyme for genome duplication, with its large subunit supplying catalytic activity and the three other subunits serving structural roles [1, 2]. While the best understood role for Pol ε is in leading strand synthesis, additional functions in replisome assembly, replication checkpoint activation, and chromatin assembly have been described [3–7]. How these dynamic functions of Pol ε are post-translationally regulated has been largely unclear.

We reported previously that the large subunit of Pol ε in budding yeast, namely Pol2, is sumoylated, though details of this modification and its effects have been elusive [8]. In general, conjugation of SUMO (small ubiquitin like modifier) onto lysine residue(s) has been shown to alter substrate properties, such as interactions with other proteins, stability, and activities [9]. SUMO modification tends to be highly dynamic due to the interplay of SUMOylation enzymes and SUMO proteases in cells [10]. The dynamic nature of this modification makes it well-suited to the rapid modulation of substrate function in response to environmental changes. A wide spectrum of effects has been reported for SUMO modification, from the fine-tuning of substrate attributes to binary switch-type regulation [11]. While the organisms examined thus far contain a single SUMO E1 and E2, multiple E3s (ligases) exist to increase substrate specificity. Budding yeast SUMO E3s include the homologous Siz1 and Siz2 proteins and the Mms21 subunit of the Smc5/6 complex [12, 13]. These E3s are conserved from yeast to humans and have been broadly implicated in genome maintenance. In particular, mutations of the human Smc5/6 complex, including its SUMO E3 subunit, have been linked to genome instability syndromes [14, 15].

Sumoylation has been recently implicated in regulating DNA replication, but important details underlying this regulation remain outstanding [16]. Only a few of the many sumoylated DNA replication factors found in yeast and humans have been examined. In the best-known example, Siz-mediated sumoylation of the DNA polymerase clamp PCNA disfavors recombinational repair [17, 18]. Another recent example describes the importance of MCM replicative helicase sumoylation during G1 phase for prevention of premature replication initiation [19]. Whether sumoylation plays direct role(s) in replication elongation has been unclear.

We examined Pol2 sumoylation in yeast to better understand how sumoylation may modulate replication. We mapped the Pol2 sumoylation site to a single lysine residue within this large protein of over two thousand amino acids. Interestingly, the Pol2 sumoylation site is located within an insertion of the catalytic domain far from the active site. This insertion is highly conserved among Pol2 orthologs, but absent in all other replicative polymerases, suggesting that it contributes to unique Pol ε function(s). We provide several lines of evidence to suggest that Pol2 sumoylation positively regulates replication fork progression and helps minimize gross chromosomal re-arrangements.

## Results

### Pol2 sumoylation occurs at a single lysine within its catalytic domain

**Fig 1:**
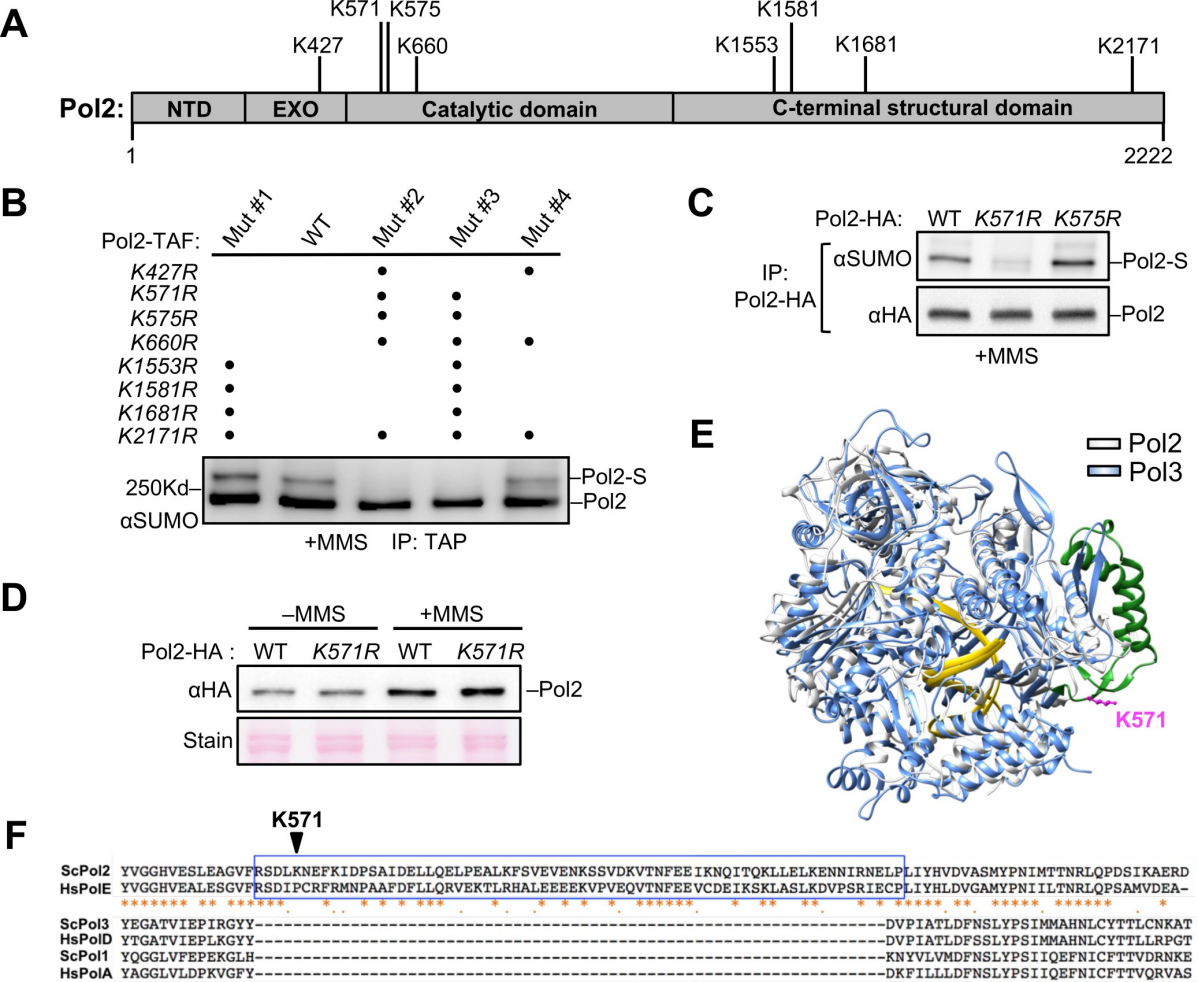
Pol2 is sumoylated at a lysine residue within an insertion in its catalytic domain. **(A)** Schematics of Pol2 protein domains and candidate sumoylation sites. The Pol2 domains depicted include its N-terminal domain (NTD), exonuclease domain (EXO), catalytic domain, and C-terminal structural domain. Eight lysine residues fitting within the sumoylation consensus motif are labeled. **(B)** Sumoylation of wild-type and mutant Pol2 proteins. TAF-tagged Pol2 was immunoprecipitated and examined by Western blotting using anti-SUMO antibody. In wild-type (WT) cells, the Pol2 sumoylated form (Pol2-S) was detected as a band migrating above the unmodified form (Pol2). Detection of the unmodified form arises from the interaction of the nonspecific region of the antibody with the Protein A (ProA) portion of TAF, as elucidated previously [8]. In each of the *pol2* mutant constructs, dots indicate the lysine residues mutated to arginine. **(C)** Sumoylation of Pol2 is largely abolished by mutating lysine 571, but not lysine 575. Experiments were done as in panel (A), except that HA-tagged Pol2 was examined and immunoblots were first probed with an anti-HA antibody to detect the unmodified form of Pol2. Due to the low level of Pol2 sumoylation, the sumoylated Pol2 form was not visible at the exposure shown when probing with anti-HA antibody but was detectable using anti-SUMO antibody. In all figure panels when proteins were examined, representative Western blots of two or more biological replicates are shown. **(D)** The *pol2* sumoylation mutant does not affect protein levels. Total protein extracts were examined during normal growth (-MMS) and after MMS treatment (+MMS). Stain indicates equal loading levels. **(E)** Overlay of the catalytic domain structures from the budding yeast Pol2 and Pol3. The crystal structure of Pol2 catalytic domain (cornflower blue) (PDB: 4M8O) is superimposed upon that of Pol3 (light grey) (PDB: 3IAY). DNA is indicated in gold. The insertion containing the Pol2 sumoylation site is colored green and K571 is colored pink. **(F)** Sequence alignments of the insertion containing the Pol2 sumoylation and adjacent regions among replicative polymerases. These regions from Pol2 (ScPol2) and human POLE (HsPOLE) are boxed blue and absent in the catalytic subunits of DNA polymerase α (ScPol1 and HsPolA) and δ (ScPol3 and HsPolD). Adjacent regions share homology amongst all polymerases. Asterisks and dots label conserved and similar residues, respectively.

The first step we took towards understanding the function of Pol2 sumoylation was identifying the modification site. Recent proteomic data found that nearly half of all sumoylation events occur at the sumoylation consensus motif, ψKxE/D, where ψ is a large hydrophobic residue [20–22]. We thus examined eight lysine residues in Pol2 located within such a motif (Fig 1A). These residues are distributed over multiple domains of Pol2 (Fig 1A), including the proof-reading exonuclease domain, the DNA polymerase domain, and a large C-terminal structural domain important for binding to other Pol ε subunits and for the DNA replication checkpoint response.

As an initial screening strategy, we generated four Pol2 constructs, each containing arginine substitutions at several lysine residues of interest. To better detect Pol2 sumoylation, cells were treated with methyl methanesulfonate (MMS), a replication stress agent known to upregulate this modification [8]. We used an established immunoprecipitation method that preserves post-translational modifications of a target protein while preventing co-purification of associated proteins [8]. As shown previously, a single sumoylated form of TAF (ProA-Flag)-tagged wild-type Pol2 was detected on immunoblots using a SUMO-specific antibody (Fig 1B) [8]. Two of the tested *pol2* mutant constructs abolished this sumoylated form (Fig 1B). We reasoned that four lysine residues mutated on both constructs could be responsible for Pol2 sumoylation. Among these, K660 and K2171 were excluded from further analyses as their mutations were also present in constructs that did not reduce Pol2 sumoylation levels (Fig 1B); thus, only K571 and K575 were further examined.

To avoid potentially adverse effects that may be associated with a large tag such as TAF, we examined endogenous Pol2 fused to an HA tag lacking lysine residues. We found that Pol2-HA sumoylation was greatly reduced by *K571R*, but not *K575R* (Fig 1C), suggesting that K571 was responsible for the bulk of Pol2 sumoylation. We verified that *pol2-K571R* (*pol2-KR*) did not affect Pol2 protein levels before or after MMS treatment (Fig 1D).

The sumoylation site identified above is located within the Pol2 catalytic domain. This domain closely resembles the catalytic domains of other DNA polymerases at both sequence and structural levels [23]. However, K571 is situated within a sixty-six amino acid insertion that is only present in Pol2-family members (Fig 1E and 1F) [24]. This insertion is located at the periphery of the catalytic domain, far from its DNA binding and active sites, rendering it accessible for modification (Fig 1E) [24]. Though the role of this insertion is unclear, its high level of sequence conservation suggests functional importance (Fig 1F). Thus, our data suggests that Pol2 sumoylation mainly occurs at a lysine located within a unique catalytic domain insertion.

### Upregulation of Pol2 sumoylation upon replication fork stalling depends on the Mms21 SUMO ligase and the Mec1 kinase

**Fig 2:**
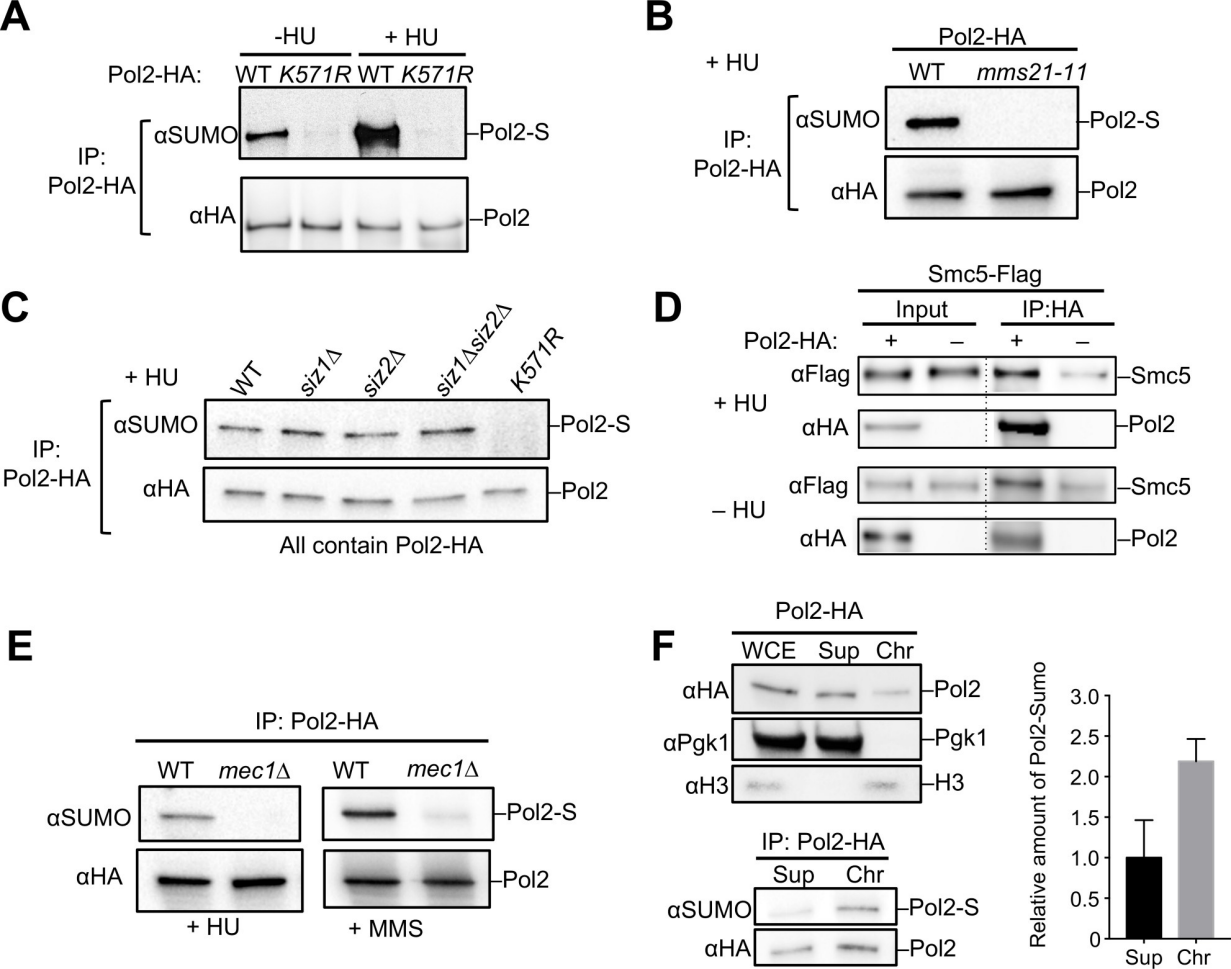
Pol2 interacts with the Smc5/6 complex, and its sumoylation is dependent on this complex and Mec1 under genotoxic conditions. **(A)** Pol2 sumoylation is induced by HU treatment. Cells were treated with HU (+HU) or without HU (-HU) and examined for Pol2 sumoylation as in Fig 1C. In both situations, *pol2-K571R* abolished Pol2 sumoylation. **(B)-(C**) Pol2 sumoylation is abolished by mutation of the Mms21 but not Siz1/2 SUMO E3s. Experiments were performed as described for panel (A). (**D**) Smc5 and Pol2 associate with each other *in vivo*. Immunoprecipitation of HA-tagged Pol2 co-purifies Flag-tagged Smc5 in both HU treated and non-treated cells. The control (–) shows that Smc5 exhibits a low level of bead-binding when Pol2 is untagged. (**E)** Mec1 is required for Pol2 sumoylation under HU and MMS conditions. Experiments were performed as described in panel (A) for HU conditions and in Fig 1C for MMS conditions. The viability of *mec1Δ* cells was maintained by *sml1Δ*, which does not affect Mec1-mediated checkpoint functions [27]. (**F)** A major population of sumoylated Pol2 is associated with chromatin. Left top: Whole cell extract (WCE), chromatin fraction (Chr), and soluble fraction (Sup) were examined by Western blotting. H3 and Pgk1 were used as markers for the chromatin and non-chromatin fractions, respectively. Left bottom: HA-tagged Pol2 was immunoprecipitated from chromatin-bound and soluble fractions and examined as in panel (A). Right: The relative levels of sumoylated vs. unmodified Pol2 in both fractions were plotted based on experiments using two different spore clones for each genotype.

Our finding that the catalytic domain of Pol2 is sumoylated suggests a potential regulatory role for this modification in DNA synthesis. To test this idea, we first examined the effect of DNA synthesis blockade on Pol2 sumoylation. Pol2 sumoylation levels are known to increase upon MMS treatment, which generates template lesions that impede DNA synthesis [8]. We also treated cells with hydroxyurea (HU), which impairs DNA synthesis by slowing down replication forks. We detected robust upregulation of Pol2 sumoylation after HU treatment (Fig 2A). Significantly, as for MMS conditions, Pol2 sumoylation in HU or during normal growth also depends on K571 (Fig 2A). Thus, Pol2 sumoylation at the same residue is upregulated when replication fork progression is impaired by either MMS or HU treatment.

We went on to ask which SUMO E3(s) are responsible for Pol2 sumoylation in HU conditions. Pol2 sumoylation was eliminated by mutating the SUMO E3 domain of the Mms21 subunit of the Smc5/6 complex (*mms21-11*), which is known to promote replication fork progression (Fig 2B) [12, 25]. In contrast, removal of either or both of the Siz SUMO E3s did not affect Pol2 sumoylation (Fig 2C). Co-immunoprecipitation experiments further demonstrated that Pol2 is associated with Smc5/6 in the presence or absence of HU (Fig 2D), supporting the proximity between this E3 complex and Pol2. As the Mec1 checkpoint kinase also positively affects replication fork progression in HU and MMS conditions [26], we tested the effect of *mec1* mutation. Pol2 sumoylation was absent in *mec1Δ* cells upon either HU or MMS treatment (Fig 2E), suggesting that Mec1 contributes to Pol2 sumoylation when DNA synthesis is impaired by genotoxins.

The observations above link Pol2 sumoylation to replication fork blockade and to factors involved in overcoming such blockade. We next asked whether the sumoylated form of Pol2 is associated with chromatin. To this end, we performed chromatin fractionation followed by immunoprecipitation of Pol2. The Pol2 sumoylated form was found to be enriched more than two-fold in the chromatin fraction relative to the soluble fraction (Fig 2F). That a major fraction of sumoylated Pol2 is associated with DNA supports involvement of this modification in DNA synthesis.

### S-phase-specific Pol2 sumoylation requires replication initiation and increases when replication fork progression is impaired

**Fig 3:**
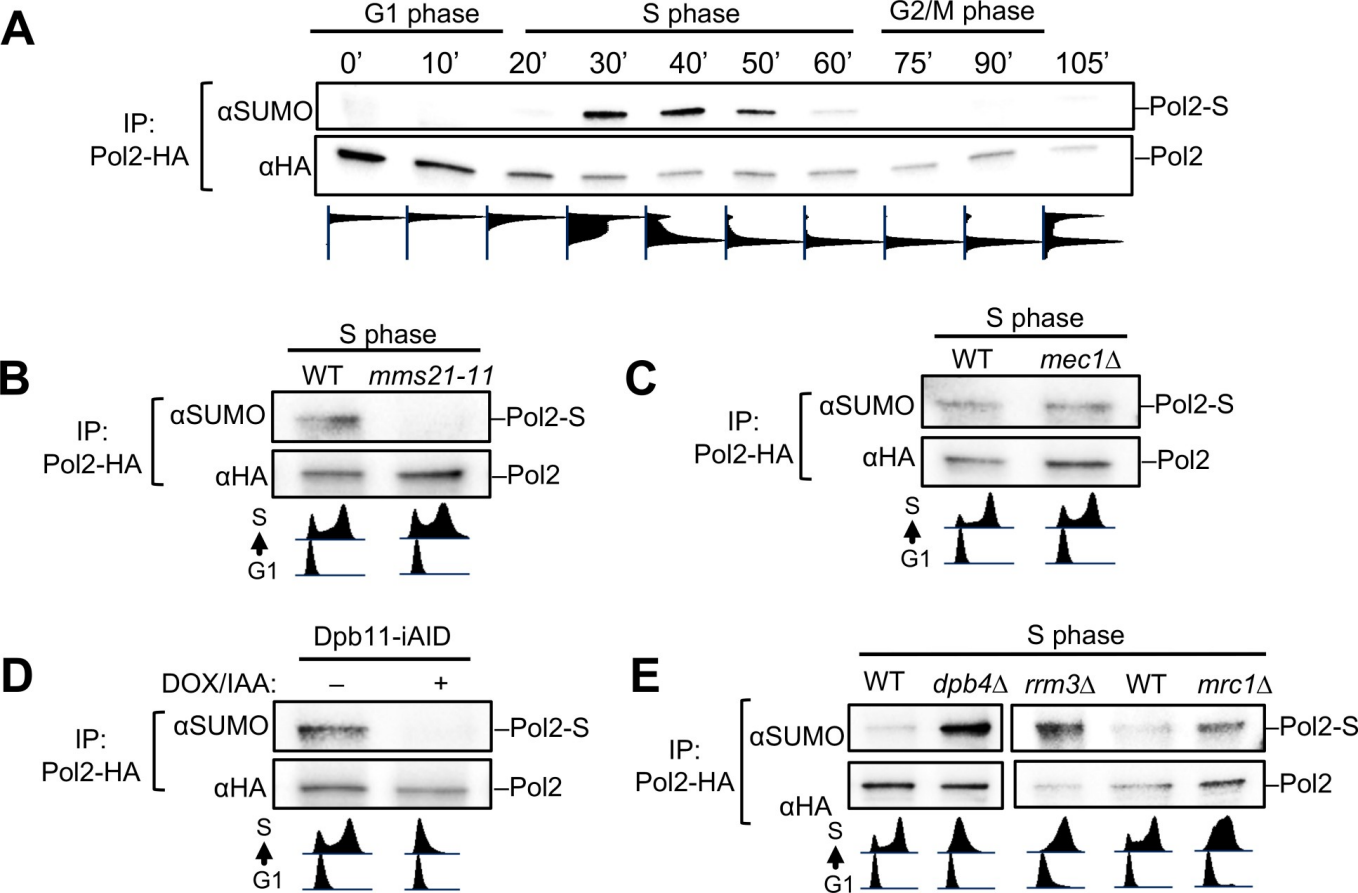
S phase-specific Pol2 sumoylation and its genetic determinants. **(A)** The temporal pattern of Pol2 sumoylation throughout the cell cycle. Wild-type cells containing Pol2-HA were arrested in G1 and then released into the cell cycle. Pol2 sumoylation was examined at the timepoints indicated, as in Fig 2A. Cell cycle progression was monitored by flow cytometry (Bottom). (**B)-(C)** Pol2 sumoylation in normal S phase requires Mms21, but not Mec1. As in panel (A), cells were examined after G1 cells had progressed into S phase. Note that *mec1Δ* cells contain *sml1Δ* to sustain viability. (**D)** Pol2 sumoylation requires the replication initiation factor Dpb11. Cells containing iAID-degron-tagged Dpb11 were analyzed as described for panel (A). As shown previously [56], cell became defective in replication initiation upon the addition of doxycycline (Dox), which turns off Dpb11-iAID expression, and IAA, which degrades Dpb11-iAID fusion proteins. (**E**) Pol2 sumoylation increases in cells lacking Dpb4, Rrm3, or Mrc1. Experiments were done, and data is presented as described for panel (B). The reduction of Pol2 sumoylation levels was based on experiments using two different spore clones for each genotype.

Our results so far suggest a role for Pol2 sumoylation in replication fork progression. To test this premise further, we examined the temporal pattern of Pol2 sumoylation during normal growth. When cells were synchronized in G1 and released into the cell cycle, we found that Pol2 sumoylation occurred specifically in S phase: Pol2 sumoylation was minimal in G1 and G2/M cells but became clearer in early S phase (20 min) and stronger in mid- and late-S phase cells (30–50 min) (Fig 3A). We confirmed that Pol2 sumoylation depends on Mms21 during normal S phase as much as under the HU conditions above (Fig 3B). In unstressed conditions, Mec1 is not required for DNA synthesis, notably, this is also true for Pol2 sumoylation (Fig 3C) [27]. Thus, Mec1-dependent Pol2 sumoylation occurs specifically in response to replication stress when Mec1 is required for replication elongation.

We next tested whether S phase-specific Pol2 sumoylation requires replication initiation. To this end, we acutely depleted origin firing factor Dpb11 in G1 phase before cells were released into the cell cycle. Dpb11 plays a structural role in replisome assembly during replication initiation, but does not travel with the replisome, so would not be expected to directly affect fork progression [4]. We found that Dpb11 loss eliminates Pol2 sumoylation in S phase (Fig 3D). We further queried Pol2 sumoylation level changes with 3 loss-of-function mutations known to reduce fork progression: 1) the Dpb4 subunit of Polε, 2) the Rrm3 helicase that strips off template barriers, or 3) the Mrc1 protein that promotes fork speed [28–31]. We found that Pol2 sumoylation levels increased in all three situations relative to wild-type cells (Fig 3E). The opposing effects of impaired replication initiation vs. impaired elongation on Pol2 sumoylation suggest that bulk Pol2 sumoylation occurs after replication initiation and is stimulated when fork progression is impeded.

### *pol2-KR* sensitizes another Pol ε mutant during normal growth and in replication stress.

**Fig 4:**
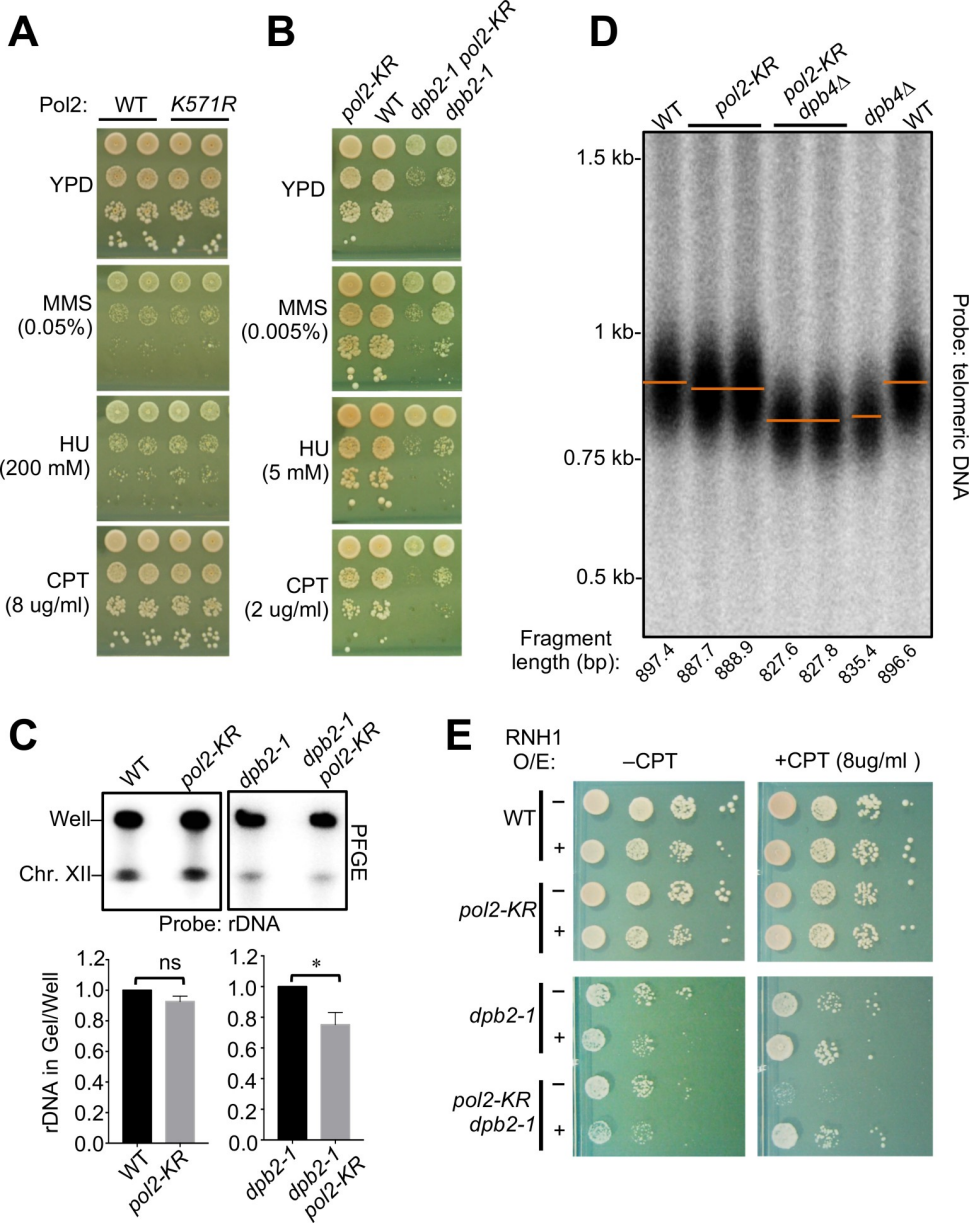
The *pol2-KR* sensitization phenotype reflects a defect in replication fork progression. **(A)** *pol2-KR* cells exhibit no overt growth defects or genotoxic sensitivities. Two spore clones of each genotype were examined; cells were spotted in 10-fold serial dilutions on plates containing no drug (YPD) or indicated concentration of drugs. **(B)** *pol2-KR* is synthetically sick with *dpb2-1*. As in panel (A), results of a set of representative spore clones are shown. **(C)** Examination of Chr XII replication. S phase cells were examined by PFGE followed by Southern blotting with a probe specific to the rDNA locus on Chr XII. The relative levels of Chr XII signals in the gel and in the wells were quantified from two biological duplicates; means and SDs are plotted. Statistical significance is derived by Student’s t-test (* indicates *p* <0.05; ns = not statistically significant). **(D)** Cells unable to sumoylate Pol2 harbor shorter telomeres. Genomic DNA was digested using XhoI and PstI and separated on gels. Telomere and the associated 600-bp Y’ sub-telomere fragments were detected using telomere-specific probes. The mid-point of each fragment is indicated by a line and was used to deduce fragment size as marked under the blot (see method) **(E)** The CPT sensitivity of *pol2-KR*, *dpb2-1* cells is suppressed by RNH1 overexpression. Cells with (+) and without (–) a Gal-inducible RNaseH1 enzyme were spotted at 10-fold serial dilution on plates containing galactose with or without CPT.

A role for Pol2 sumoylation in fork progression is strongly suggested by both its temporal-spatial patterns and genetic dependencies. To further understand this connection, we examined the consequences of Pol2 sumoylation loss. The growth of *pol2-KR* cells was indistinguishable from that of wild-type cells in the presence and absence of replicative stress agents (Fig 4A). However, *pol2-KR* exhibited synthetic defects with a mutant of the Pol ε subunit Dpb2 (Fig 4B). Dpb2 is the only other essential subunit in Pol ε besides Pol2 and binds to a Pol2 C-terminal structural region; *dpb2-1* interferes with this interaction and retards growth [32–34]. *pol2-KR* sensitizes *dpb2-1* during growth and under treatment with the replication blocking agents HU, MMS and topoisomerase 1 (Top1) inhibitor camptothecin (CPT) (Fig 4B). This data again supported a positive effect of Pol2 sumoylation on DNA replication, and more significantly, a role that could be compensated for by another Pol ε subunit.

### *The pol2-KR* phenotype suggests a role for Pol2 sumoylation in fork progression

We reasoned that the negative interaction of *pol2-KR* and *dpb2-1* may be due to additive impairment of DNA synthesis. To test this idea, we first assessed chromosome XII (Chr XII) synthesis. Chr XII is a sensitive indicator of dysregulated replication since it harbors the largest burden of endogenous replication fork barriers in its ribosomal DNA (rDNA) locus, such as RNA-DNA hybrids (R-loops) and protein barriers [30, 35, 36]. We examined synchronized S phase samples using pulsed field gel electrophoresis (PFGE), which separates fully replicated chromosomes that enter the gel from incompletely replicated branched forms trapped in wells. We probed chromosomes separated by PFGE using a probe specific to Chr XII to calculate the ratio of Chr XII signals from gel bands versus signals in the well. We found that *pol2-KR* did engender additional Chr XII synthesis defects in *dpb2-1* cells, since *pol2-KR dpb2-1* double mutant cells showed an approximately 25% reduction in replicated Chr XII than *dpb2-1* single mutant cells (Fig 4C). These data suggest that Pol2 sumoylation is required for the optimal synthesis of a difficult-to-replicate chromosome when Pol ε function is suboptimal.

In light of the above results, we examined telomeric regions that are also challenging to replicate due to high levels of R-loops and other types of barriers [37]. As defective telomeric replication leads to shorter telomeres, we measured telomere length by Southern blot. We found that average telomeres sizes in *pol2-KR* mutant were reproducibly reduced about 10 bp, a statistically significant difference (Fig 4D; see method). A similar reduction was also seen in the *dpb4Δ* background, which itself led to moderately shorter telomeres (Fig 4D). These data suggest that Pol2 sumoylation and Dpb4 contribute to telomere synthesis by non-overlapping mechanisms.

One shared feature of rDNA and telomeric regions is R-loop enrichment [38, 39]. To address whether *pol2-KR* defects in replication are related to this type of replication blockade, we examined *pol2-KR* cells after Top1 inhibition, which increases R-loop levels [40–42]. The *pol2-KR dpb2-1* double mutants were sensitive to the Top1 inhibitor CPT, and this sensitivity was suppressed by overexpressing RNase H1, which removes RNA-DNA hybrids. This result suggests that *dpb2* mutant cells divested of Pol2 sumoylation are less capable of coping with CPT, likely because of their reduced ability to overcome R-loop-mediated fork blockade.

### Pol2 sumoylation limits recombinational repair and genomic alteration

**Fig 5:**
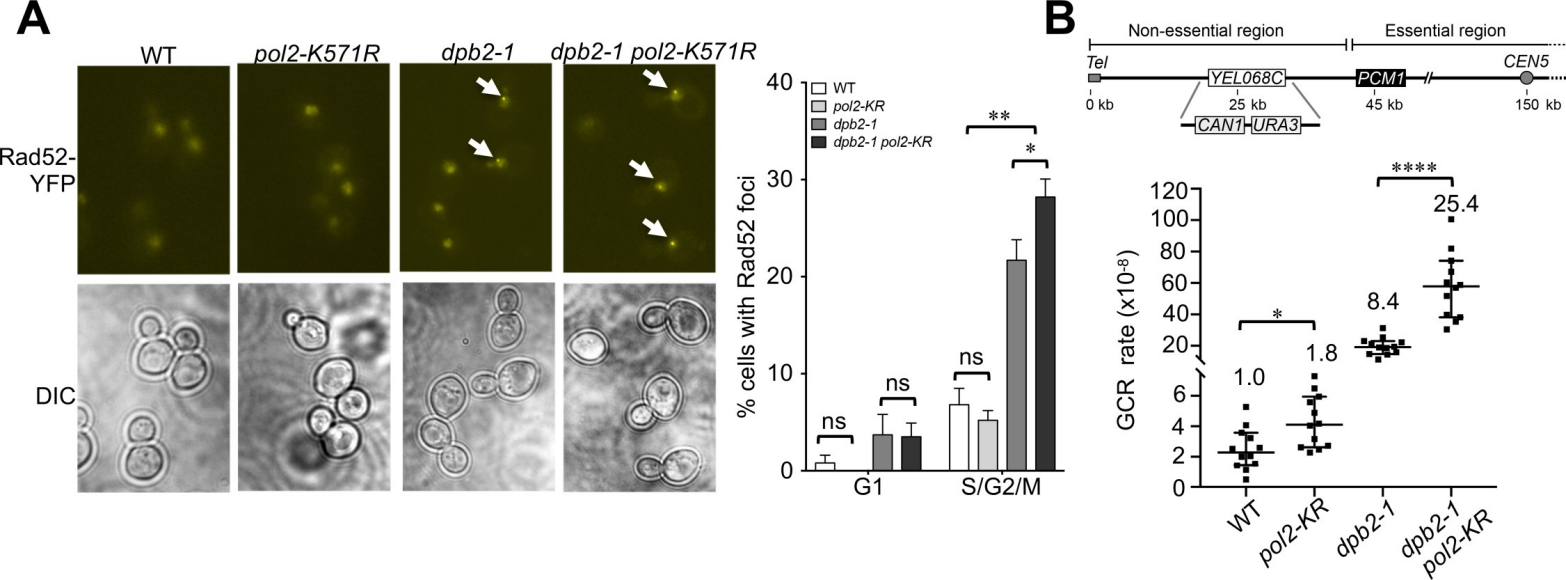
*pol2-KR* increases Rad52 foci and GCR levels in *dpb2-1* cells. **(A)** Examination of Rad52 foci levels. Cells of the indicated genotypes contained YFP-fused Rad52 at its endogenous locus. Left: Representative YFP and DIC images. White arrows indicate Rad52-YFP foci present in the background of diffuse nuclear Rad52-YFP signals as seen previously [57]. Right: Quantification of the percentage of cells containing Rad52-YFP foci. Statistical significance was determined by Chi-Square test (* indicates *p*<0.05 and ** indicates *p*<0.01). **(B)** Examination of GCR rates. Top: Schematic of the GCR assay as described [58]. Bottom: GCR rates for the strains indicated. Dot plot displays all data points collected for nine to twelve cultures from two biological replicates per genotype. The median and 95% confidence interval were indicated by a horizontal line and errors, respectively. Two-tailed Mann-Whitney test was performed to determine statistical significance. *, p<0.05; ****, p<0.0001.

Our results above support a role for Pol2 sumoylation in fork progression through R-loops. As replication fork blockade by R-loops leads to increased recombinational repair and genome rearrangements [43], our model predicts that loss of Pol2 sumoylation will increase these events. We first examined repair foci formed by the recombination factor Rad52, as previously described [44]. We found that although the Pol2 sumoylation mutant *pol2-K571R* did not by itself alter Rad52 foci levels during growth, it increased Rad52 foci levels in budded cells by about 30% in the *dpb2-1* background (Fig 5A). Next, we assayed genomic alteration using the gross chromosomal re-arrangement (GCR) assay that simultaneously assesses loss of two markers at a nonessential region of chromosome V (Fig 5B, top) [45]. We found that the *pol2-KR* allele alone led to a nearly two-fold increase in GCR rates, and that it was additive with *dpb2-1* such that their double mutants exhibited a three-fold increase in GCR rates over that of the *dpb2-1* mutant (Fig 5B). Taken together, these data support a role for Pol2 sumoylation in restraining GCRs and recombinational repair.

## Discussion

We describe the spatial and temporal regulation of Pol2 sumoylation, its genetic requirements, and its functions and effects on genome stability. Collectively, our data suggest that Pol2 sumoylation is tightly regulated, associated with DNA synthesis, and can contribute to replication fork progression. Furthermore, our findings link Pol2 sumoylation with Smc5/6-mediated roles in genome synthesis. This study also broadens our understanding of the effects of sumoylation on DNA replication by suggesting that SUMO can directly influence fork progression by regulating the leading strand polymerase.

Several cohesive lines of evidence support that Pol2 sumoylation positively affects replication fork progression. The observation that Pol2 sumoylation is S-phase-specific (Fig 3A) and requires replication initiation factor Dpb11 (Fig 3D) suggests that bulk Pol2 sumoylation arises after the replisome has been assembled. The observation of increased Pol2 sumoylation levels upon inhibition of fork progression by genotoxins or loss of factors required for optimal fork movement strengthens the link to elongation (Figs 2A and 3E). This link was also supported by a Mec1 requirement for Pol2 sumoylation only in situations where Mec1 is required for replication fork progression (Figs 2E and 3C).

Moreover, the Smc5/6 SUMO ligase, which is known to promote genome duplication, binds Pol2 and is responsible for its sumoylation (Figs 2B, 2D and 3B), suggesting that Pol2 sumoylation partly accounts for Smc5/6’s contribution to replication. A positive effect of Pol2 sumoylation on replication elongation is further supported by the genetic interaction between *pol2-KR* and *dpb2-1* when assaying for replication of a difficult-to-replicate chromosome and survival under conditions enhancing R-loop-mediated replication stress (Fig 4B and 4C). We note that the observed Chr XII replication defects most likely stem from difficulty in duplicating rDNA, whose replication status is checkpoint-blind [46], though direct tests of rDNA replication will be needed to verify this conclusion. Interestingly, we found that Rnh1 overexpression relieved the *pol2-KR* sensitizing effect on *dpb2-1* under R-loop-mediated replication stress (Fig 4E). Considering our other findings described above, the simplest interpretation of this suppression is that Pol2 sumoylation can be helpful for coping with R-loop levels during replication elongation. This conclusion is also consistent with a requirement for Pol2 sumoylation in maintaining wild-type telomere length (Fig 4D).

Collectively, our data suggest that Pol2 sumoylation contributes to replication fork progression under conditions of both endogenous and exogenous fork blockade. Though the underlying mechanisms of such a role require future examination, sumoylation site localization to the Pol2 catalytic domain suggests a direct effect in modulating DNA synthesis (Fig 1A and 1C). This model is further supported by the observation that the majority of sumoylated Pol2 is chromatin-bound during replication (Fig 2F). As K571 is located inside a conserved insertion unique to Pol2 family members, its sumoylation likely affects Pol2 family-specific functions, such as processive leading strand synthesis. Based on the fact that this insertion lies at the periphery of the catalytic domain and far from the Pol2 DNA-binding and polymerization site in the crystal structure (Fig 1E) [24], sumoylation of K571 is less likely to influence Pol ε catalytic activity directly. We also excluded an effect of sumoylation on overall Pol2 stability (Fig 1D). Complementary work from the De Piccoli lab suggests that Pol2 sumoylation at K571 is linked to its ability to bind SUMO, so it is plausible that sumoylation aids Pol2 function through modulation of its protein-protein interactions. For example, Pol2 sumoylation and SUMO binding may collaborate in the recruitment of additional replisome-stabilizing factors to stalled forks, such as those known to contact the Pol2-NT region, including Mrc1, Ctf18, and Cdc45. The distinct effects exerted by Mec1 and by the examined replication factors on Pol2 sumoylation, as well as the temporal pattern of modification, also support Pol2 sumoylation in the context of an assembled replisome. However, our data do not exclude the possibility that Pol2 sumoylation may also affect additional roles of Pol ε.

The phenotype of the Pol2 sumoylation mutant *pol2-K571R* by itself is mild, with ~10-bp decrease in telomere length and nearly two-fold increase in GCR rates (Figs 4D and 5B). However, *pol2-K571R* defects are compensated for by the functions of the Dpb2 subunit of Pol ε, since *pol2-K571R* is additive with *dpb2-1* in multiple genome maintenance assays. Specifically, compared to *dpb2-1* cells, the *pol2-K571R dpb2-1* double mutant shows: i) slower growth and stronger sensitivity to several replicative stress agents, ii) about 25% less chromosome XII replication, iii) nearly 30% more recombinational repair foci, and iv) approximately 3-fold higher GCR rates (Figs 4B and 4C; 5A and 5B). These observations suggest that Pol2 sumoylation contributes to, but may not be critical for, DNA replication and stability; its contributions are uncovered by sub-optimal Dpb2 function. The moderate effects of sumoylation on individual DNA repair factors have been reported and lead to the proposal that the overall strength of sumoylation-based regulation arises from the compounded effects of multiple sumoylation events, so abolishing individual sumoylation sites often does not lead to a strong phenotype [47]. Our data suggest that a similar concept may apply for SUMO in the DNA replication process, and future studies on the sumoylation of other DNA replication factors will help to address this possibility.

Finally, our findings link Pol2 sumoylation during normal S phase and under HU or MMS treated conditions to the Smc5/6 complex. Several studies have shown that Smc5/6 promotes genome duplication during growth and under replication stress [25]. Our finding adds to this paradigm by suggesting that part of Smc5/6’s effects are mediated through sumoylation of Pol2. In summary, our work reveals for the first time that Pol2 sumoylation is temporally and spatially regulated, and that it contributes to Pol2 functions in DNA synthesis, particularly at difficult-to-replicate loci. Our findings lay the foundation for a deeper understanding of Pol2 post-translational regulation, as well as the roles played by Smc5/6 and SUMO in DNA replication.

## Methods

### Yeast Strains and Genetic Manipulations

Strains used are isogenic to W1588-4C, a *RAD5* derivative of W303 (*MAT*a *ade2-1 can1-100 ura3-1 his3-11*,*15 leu2-3*,*112 trp1-1 rad5-535*) [12]. Strains and plasmids are listed in S1 Table. At least two biological replicates were performed for each experiment. Standard yeast protocols were used for protein tagging and constructions of mutant strains, medium preparation, cell growth, and spot assays. To generate *pol2* mutant strains, DNA fragments containing the desired mutations and a selection marker were generated by PCR. Upon transformation of wild-type cells with PCR fragments, colonies containing the correct gene replacements were identified by PCR and sequencing. For treating cultures with drugs, 0.3% MMS or 200mM HU was added to log phase cultures for 2 hr before harvesting. For spot assays, log phase cultures were serially diluted 10-fold and spotted onto plates containing standard yeast media, including YPD (Fig 4A and 4B) and SC-URA+Galactose (Fig 4E), either alone or with the addition of the indicated concentration of drugs. Plates were incubated at 30°C unless indicated, and photographed after 24–48 hrs.

### Detection of sumoylated proteins and other protein methods

Examination of protein sumoylation was carried out as previously described [8]. Briefly, cell lysates were prepared using bead beating methods under denaturing conditions to prevent desumoylation during protein extraction and minimize co-purification of associated proteins. Diluted protein extracts were then immunoprecipitated, using IgG-sepharose (Sigma) to pull down TAP-tagged Pol2, or Protein G-agarose plus anti-HA (12CA5) antibody to pull down HA-tagged Pol2. Immunoprecipitated proteins were washed and eluted with loading dye before separation on standard SDS-PAGE gels and immunoblotting with anti-SUMO [12] and tag-specific antibodies including Peroxidase Anti-Peroxidase (Sigma-Aldrich, P1291) and anti-HA (Sigma, 12CA5). As for most sumoylated proteins, the sumoylated form of Pol2 is of low abundance and not seen under normal exposure using anti-tag antibodies but can be readily detected by anti-SUMO antibody. Standard methods for detecting protein levels in crude cell extracts and protein interactions by co-IP were used. For experiments in Fig 2D, DNase was added to remove DNA before immunoprecipitation, and anti-Flag antibody was obtained from Sigma-Aldrich (F1804).

### Cell cycle analyses and PFGE

G1 cells were synchronized with α-factor and released into cycling after washing off α-factor. Cell cycle progression was monitored by flow cytometry as described previously [48]. For experiments in Fig 4C, agarose plugs were prepared from late S phase samples, 1% Megabase agarose gels were run on a Bio-Rad CHEF-DR III apparatus, and chromosomes were transferred and probed with rDNA-specific probe by Southern blotting as described previously [49].

### GCR assays

GCR rates measurement was performed by fluctuation analysis as described previously [50], except that the assay was applied to the same genetic background as the other strains used in this work [51]. Briefly, for each genotype, at least 9 cultures were examined in at least two different strains. Yeast cells were washed, and serial dilutions were plated on media containing canavanine and 5-FOA to select for the loss of *CAN1* and *URA3* on FC media and synthetic complex (SC) plates. GCR rates were calculated as m/NT using the following formula: m*(1.24 + ln[m])–NFC = 0. m: mutational events, NFC: numbers of colonies on FC plates, NT: numbers of colony on SC plates. The upper and lower 95% confidence intervals (95% CI) were calculated as described [50]. For statistically analysis, the two-tailed Mann-Whitney test was performed as described previously [52, 53] using GraphPad Prism version 7.

### Other Methods

Telomere length was measured by Southern blotting as previously described [54], using XhoI and PstI to digest genomic DNA and a C_1–3_A/ TG_1–3_ telomere DNA probe to detect telomere repeats. Telomere blots were analyzed using the TeloTool software [55] to determine the mid-points the telomere fragments that are associated with 600-bp Y’ elements, and the corresponding distances from gel wells. A calibration curve using DNA molecular markers was made to correlate the distances of the DNA bands from the well with their sizes. After fitting to this curve, the average sizes of and standard deviation of the fragments were determined. The telomere sizes were further calculated by subtracting the 600-bp Y’ sequences from the detected fragment sizes and are 297.0 ± 0.6 bp for wild-type cells and 288.3 ± 0.8 bp for *pol2-KR* cells, respectively. Student t-test showed that the difference between wild-type and *pol2-KR* telomere lengths are statistical significance (p = 0.007). For measuring Rad52 foci, cells were imaged on an Axioimager microscope with 100X objective lens (NA = 1.4). DIC and YFP images were captured at 14–18 Z-sections with a 0.5 mm step size to cover the whole yeast cell; maximal projections are shown. Exposure time for Rad52-YFP was 1s. Student’s t-tests were used for statistical analysis unless indicated otherwise.

## Supporting information

S1 Table Strains and plasmids used in this study. Only one strain is listed for each genotype, but at least two independent isolates of each genotype were used in the experiments.
(DOCX)
