# Supplementary material for: Sumoylation of the DNA polymerase ε by the Smc5/6 complex contributes to DNA replication
Source: PLoS Genet. 2019 Nov 25;15(11):e1008426. doi: 10.1371/journal.pgen.1008426 (PMC6876774; doi:10.1371/journal.pgen.1008426)
Supplement: S1 Table — Only one strain is listed for each genotype, but at least two independent isolates of each genotype were used in the experiments. (DOCX) [file pgen.1008426.s001.docx]

| **Strain** | **Genotype** | **Source** |
| --- | --- | --- |
| T887 | *Mat alpha pol2-K1553R K1581R K1681R K2171R-TAF::KAN* | This study |
| X3603-2d | *Mat alpha pol2-K427R K571R K575R K660R K2171R-TAF::KAN* | This study |
| T892 | *Mat alpha pol2-K571R K575R K660R K1553R K1581R K1681R K2171R-TAF::KAN* | This study |
| T695-12 | *Mat alpha pol2-K427R K660R K2171R-TAF::KAN* | This study |
| T1001 | *Mat alpha pol2-K571R-3HA::KAN* | This study |
| T1004 | *Mat alpha pol2-K575R-3HA::KAN* | This study |
| X7021-4a | *Mat a POL2-3HA::KAN* | This study |
| X8128-6c | *Mat a mms21-11::URA3 POL2-3HA::KAN* | This study |
| X8057-3c | *siz1∆::KAN POL2-3HA::KAN* | This study |
| X8057-16c | *siz2∆::URA3 POL2-3HA::KAN* | This study |
| X8057-8c | *siz1∆::KAN siz2∆::URA3 POL2-3HA::KAN* | This study |
| X4427-7A | *Smc5-AID111-3FLAG::HIS3 ura3-1::ADH1-OsTIR1-9Myc::URA3 Pol2-3HA::LEU* | This study |
| X8124-16a | *Mat a mec1∆::TRP1 sml1∆::HIS3 POL2-3HA::KAN* | This study |
| X7981-1a | *Mat a SLD2-10FLAG::LEU2 SSN6::pST1760(HIS3) dpb11-iAID::KAN POL2-3HA::KAN* | This study |
| X8127-8d | *Mat a dpb4∆::KAN POL2-3HA::KAN* | This study |
| Z423e | *Mat a pol2-K571R* | This study |
| X7166-5b | *Mat a dpb2-1* | This study |
| X7382-1a | *Mat a pol2-K571R dpb2-1* | This study |
| X6277-6d | *dpb4∆::KAN pol2-K571R* | This study |
| X8068-6c | *ADE2 RAD52-YFP* | This study |
| X8068-2d | *pol2-K571R ADE2 RAD52-YFP* | This study |
| X8098-3c | *dpb2-1 ADE2 RAD52-YFP* | This study |
| X8098-18c | *pol2-K571R dpb2-1 ADE2 RAD52-YFP* | This study |
| X8058-11a | *ura3∆ can1Δ YEL072W::URA3-CAN1* (GCR assay strain) | This study |
| X8058-14a | *pol2-KR ura3∆ can1Δ YEL072W::URA3-CAN1* (GCR assay strain) | This study |
| X8064-6a | *dpb2-1 ura3∆ can1Δ YEL072W::URA3-CAN1* (GCR assay strain) | This study |
| X8066-2c | *pol2-K571R dpb2-1 ura3∆ can1Δ YEL072W::URA3-CAN1* (GCR assay strain) | This study |
| X8144-9c | *Mat a POL2-3HA::KAN* *rrm3∆::KAN* | This study |
| X8149-5d | *Mat a POL2-3HA::KAN* *mrc1∆::TRP1* | This study |
|  |  |  |
| **Plasmid** | **Genotype** | **Source** |
| p581 | *pBY011-RNH1-URA* | [1] |

Reference

1. Makharashvili N, Arora S, Yin Y, Fu Q, Wen X, Lee J-H, et al. Sae2/CtIP prevents R-loop accumulation in eukaryotic cells. Elife. 2018;7:e42733.
